# Traffic-Related Air Pollution and Childhood Asthma: Recent Advances and Remaining Gaps in the Exposure Assessment Methods

**DOI:** 10.3390/ijerph14030312

**Published:** 2017-03-17

**Authors:** Haneen Khreis, Mark J. Nieuwenhuijsen

**Affiliations:** 1 Centre for Research in Environmental Epidemiology (CREAL), ISGlobal, 08003 Barcelona, Spain; mark.nieuwenhuijsen@isglobal.org; 2 Universitat Pompeu Fabra (UPF), 08002 Barcelona, Spain; 3 CIBER Epidemiología y Salud Pública (CIBERESP), 28029 Madrid, Spain; 4 Institute for Transport Studies, University of Leeds, LS2 9JT Leeds, UK

**Keywords:** asthma, childhood, traffic-related air pollution, exposure assessment, systematic review

## Abstract

*Background*: Current levels of traffic-related air pollution (TRAP) are associated with the development of childhood asthma, although some inconsistencies and heterogeneity remain. An important part of the uncertainty in studies of TRAP-associated asthma originates from uncertainties in the TRAP exposure assessment and assignment methods. In this work, we aim to systematically review the exposure assessment methods used in the epidemiology of TRAP and childhood asthma, highlight recent advances, remaining research gaps and make suggestions for further research. *Methods*: We systematically reviewed epidemiological studies published up until 8 September 2016 and available in Embase, Ovid MEDLINE (R), and “Transport database”. We included studies which examined the association between children’s exposure to TRAP metrics and their risk of “asthma” incidence or lifetime prevalence, from birth to the age of 18 years old. *Results*: We found 42 studies which examined the associations between TRAP and subsequent childhood asthma incidence or lifetime prevalence, published since 1999. Land-use regression modelling was the most commonly used method and nitrogen dioxide (NO_2_) was the most commonly used pollutant in the exposure assessments. Most studies estimated TRAP exposure at the residential address and only a few considered the participants’ mobility. TRAP exposure was mostly assessed at the birth year and only a few studies considered different and/or multiple exposure time windows. We recommend that further work is needed including e.g., the use of new exposure metrics such as the composition of particulate matter, oxidative potential and ultra-fine particles, improved modelling e.g., by combining different exposure assessment models, including mobility of the participants, and systematically investigating different exposure time windows. *Conclusions*: Although our previous meta-analysis found statistically significant associations for various TRAP exposures and subsequent childhood asthma, further refinement of the exposure assessment may improve the risk estimates, and shed light on critical exposure time windows, putative agents, underlying mechanisms and drivers of heterogeneity.

## 1. Introduction

Asthma is a chronic inflammatory disease of the airway which has a large impact on quality of life and poses a great burden on health services [1]. In children, asthma is the most commonly reported chronic disease in developed countries [2]. Environmental factors, importantly including improved hygiene, ambient air pollution exposures, and early-life exposures to microbes and aeroallergens, contribute to the development of asthma [2]. In a recent systematic review and meta-analyses, we found statistically significant associations between traffic-related air pollution (TRAP) and the incidence and lifetime prevalence of childhood asthma, although there was significant heterogeneity in some of the risk estimates [3]. These effects are biologically plausible. Britain’s Committee on the Medical Effects of Air Pollutants proposed four mechanisms by which air pollution can affect asthma: (1) oxidative stress and damage; (2) inflamed pathways; (3) airway remodeling; and (4) enhancement of respiratory sensitization to allergens [4]. Oxidative stress relates to common asthmatic traits [5], and was suggested to play a role in asthma pathogenesis [6]. Further, it was previously highlighted as one chief pathway which underpins the adverse health effects of (traffic-related) air pollution on the respiratory systems [7].

TRAP is a particularly important and challenging exposure to study given its ubiquity, its dominance in present urban areas, its proximity to human receptors, and its high spatial and temporal variability [8,9,10,11]. For example, the local traffic contribution to ambient nitrogen dioxide (NO_2_) can be up to 80%, and ranges between 9% and 53% for urban particulate matter less than 10 micrometres in diameter (PM_10_), and 9%–66% for urban particulate matter less than 2.5 micrometres in diameter (PM_2.5_) [8].

In the epidemiological studies included in the most recent meta-analyses of TRAP and the development of childhood asthma, different exposure assessment methods and indices have been used to characterise the exposure to TRAP, including distance to roads, active measurement of air pollutants, use of routinely measured air pollution data, land-use regression (LUR) modelling, air dispersion modelling and remote sensing [3]. These various methods and indices differ substantially and have advantages and disadvantages in terms of their spatial and temporal resolution, specificity to traffic, data and effort/expertise requirements, transferability and information provided on the actual pollutants. Furthermore, the different epidemiological studies focused on different pollutants and different exposure time windows [3]. The use of different exposure assessment methods in health effects or impacts studies can result in different estimates, partly due to the difference in accuracy and precision of the exposure estimates and the potential differential effects of different pollutants. Although the evidence base is very limited, research has shown differences, for example in the performance of and the results from dispersion models versus LUR [12,13,14] which in two studies translated into small differences in the risk estimates, but in one study translated into differences in the direction of effect estimates of NO_2_ on birth weight [13]. In a previous meta-analysis on TRAP and childhood asthma, there was some suggestion of a difference between associations with NO_2_ from within-community studies that used LUR models (five studies, odds ratio (OR) = 1.14, 95% confidence interval (CI) 1.06, 1.23) and those from studies that used dispersion models (five studies, OR = 1.02, 95% CI 0.97, 1.07) [15]. Further, there were differences in estimated health impacts when using different pollutant-specific exposure-response functions. For example, cases of asthma attributable to PM_10_ and NO_2_ differ substantially to cases of asthma attributable to black carbon [16].

In this paper, we aim to describe and discuss the exposure assessments conducted in studies of TRAP and childhood asthma development, including the methods used in the different regions, the pollutants and exposure assignment and time windows studied. We then highlight research gaps and make suggestions for further research in this rapidly growing area. Our focus is on the exposure assessments and not the effects of TRAP on asthma development per se; which we reviewed in depth elsewhere [3]. Our results and discussion are applicable to other research on TRAP and various health outcomes, beyond childhood asthma, as the exposure assessment methods are often similar [11,17].

## 2. Methods

We conducted a systematic review to synthesize the literature on TRAP exposures and the subsequent risk of childhood asthma development defined as incidence or lifetime prevalence [3]. We followed established guidance published by the University of York’s Centre for Reviews and Dissemination [18]. We registered a protocol (registration number: CRD42014015448) with the international prospective register of systematic reviews (PROSPERO) documenting our methodological approach a priori [19].

We performed the searches on 8 September 2016 via the database search interface OvidSP (http://ovidsp.ovid.com/). We searched the following databases for relevant studies: Embase (1996 to week 36, 2016), Ovid MEDLINE (R) (1996 to August 2016), and “Transport Database” (1988 to August 2016). We identified relevant studies by entering four sets of combined keywords in the “Multi-Field Search” option in OvidSP. We searched for the selected keyword combinations in “All Fields”. The keyword combinations were:

1. “Child*” AND “air pollution” AND “asthma”;
2. “Child*” AND “air quality” AND “asthma”;
3. “Child*” AND “vehicle emissions” AND “asthma”; and
4. “Child*” AND “ultra-fine particles” AND “asthma”.

We applied no limits on the initial publication date and no limits on language although we eventually excluded three foreign language studies due to translation difficulties [20,21,22]. We conducted a hand search in the reference lists of all the included studies and of previous relevant reviews we identified [15,17,23,24,25,26,27,28,29,30]. We contacted authors of unpublished studies (abstracts only) and the authors of the most recurrent studies to ensure the inclusion of all relevant published material on the topic and this resulted in the inclusion of two additional studies [31,32]. We searched Google for any other material related to “traffic-related air pollution” AND “childhood asthma” and this resulted in the inclusion of one additional study [33]. One study was also not identified in the searches but by one of the reviewers and this was included [34]. We exported studies into an Endnote X7.4 library and removed duplicates automatically using the Endnote function “Find Duplicates”. For inclusion, we selected studies that met *all* the following criteria:

- Were published epidemiological/observational studies;
- Explicitly specified the term “asthma” as an outcome for investigation;
- Examined the childhood exposure from birth until 18 years old [35] to any designated TRAP metric or established traffic-related air pollutant including proximity to roads or traffic, carbon monoxide (CO), elemental carbon (EC), nitrogen oxides (NO_x_), nitric oxide (NO), NO_2_, hydrocarbons, particles of different aerodynamic diameters (PM_2.5_, PM_10_, PM_coarse_, UFPs) or PM_2.5_ absorbance as a marker for black carbon (BC) concentrations [10,36]; and
- Examined and reported associations between preceding exposure to TRAP and subsequent risk of asthma reported as incidence or lifetime prevalence from birth until 18 years old.

All titles and abstracts were reviewed against the inclusion criteria by one researcher (Haneen Khreis) with a random 20% independently reviewed by another researcher. All potentially relevant studies were then retrieved and the available full-papers reviewed against the inclusion criteria by one researcher (Haneen Khreis) with a random 50% independently reviewed by another researcher (Mark J. Nieuwenhuijsen). Screening was undertaken manually and differences were resolved by consensus. The following data items were extracted from each included study:

1. Study reference and setting;
2. Study design;
3. Age group;
4. Number of participants;
5. Exposure assessment method(s);
6. Pollutant(s) studied;
7. Exposure assessment place;
8. Exposure assessment time; and
9. Air pollution estimates validation, if any.

Data was primarily extracted from the main papers of the included studies. Where necessary, data items were missing from the main papers, data was extracted from the supplementary materials [31,37,38,39,40,41,42,43,44,45,46,47,48,49,50,51,52], and the associated publications [53,54,55,56,57,58,59,60,61,62,63,64,65,66]. Data extraction was undertaken manually by one researcher (Haneen Khreis). A random 50% was independently reviewed by another researcher (Mark J. Nieuwenhuijsen). A fuller detail of the screening methodology can be found in Khreis et al. (2017) [3].

## 3. Results

### 3.1. Overview

**Figure 1:**
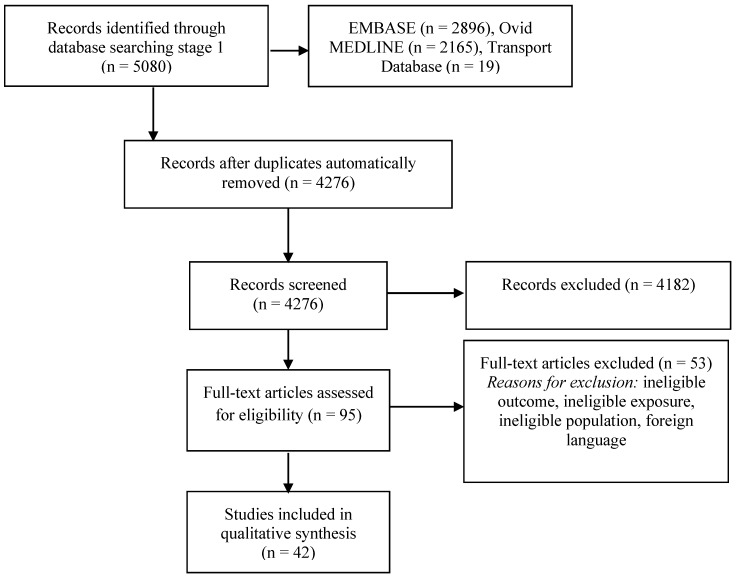
Study screening process.

The databases searches yielded 4276 unique articles, from which 95 were selected for detailed assessment of the full text, one of which was identified by a peer reviewer. Figure 1 shows the flow of papers. A total of 42 studies met our inclusion criteria [31,32,33,34,36,37,38,39,40,41,42,43,44,45,46,47,48,50,51,52,58,67,68,69,70,71,72,73,74,75,76,77,78,79,80,81,82,83,84,85,86,87] (Table 1).

A summary of the included studies’ key characteristics is shown in Table 1. Ages of participants ranged from 1 to 18 years old and sample sizes ranged from 184 [69] to 1,133,938 [85]. Follow-up periods ranged from 1 to 16 years [47]. Eighteen studies were conducted in Europe, 11 in North America, 5 in Japan, 3 in China and 1 in each of Korea and Taiwan. Thirty-two studies were cohort studies (25 of which were birth cohorts), 6 studies were case-control studies (2 of which were nested in a birth cohort), and 4 studies were cross-sectional.

### 3.2. Exposure Assessment Methods

The exposure to TRAP was assessed using different methods, sometimes in isolation and other times in combination with each other (Table 1). Most studies (*N* = 22) used LUR models, 16 studies used TRAP surrogates (e.g., proximity to roadways), 11 studies used traffic-related air pollutant concentrations measured at fixed-site monitoring stations, 8 studies used air dispersion modelling, 1 study used remote sensing and 1 study used diffusion tubes at the residence to measure NO_2_. These methods vary substantially in terms of their spatial and temporal resolution, specificity to traffic, data and effort/expertise required, transferability and information provided on the actual pollutants (Table 2). These are key criteria important in studies of TRAP and asthma (and other health effects).

In the literature, it was also apparent that the use of the different exposure assessment methods varied by region (Table 1). For example, 8 out of the 11 studies using pollutant measurements at fixed-site monitoring stations *only* used this exposure method (i.e., not in combination with other methods or metrics), 7 of which were from Japan, Taiwan, Korea and China. Also, 12 out of the 22 studies using LUR model used this exposure method *only*, 9 of which were from Europe (predominantly from the PIAMA cohort in The Netherlands), while the remaining 3 were from Canada and the USA. The remaining USA studies showed the most variability in the exposure assessment methods choice and used residential diffusion tube monitoring, dispersion modelling, fixed-site monitoring stations, proximity measures and multiple novel TRAP surrogates (see Patel et al. 2011 who used some new surrogates, including “four-way street intersection density” and “number of New York City transit bus stops”).

### 3.3. Pollutants Studied

NO_2_ was the pollutant most studied (31 studies), followed by PM_2.5_ (18 studies), BC or PM_2.5 absorbance_ (15 studies), and PM_10_ (14 studies). Other pollutants including NO_x_ (7 studies), EC (4 studies), CO (3 studies), PM_coarse_ (3 studies), NO (2 studies) were less frequently studied. Only two studies assessed particulate matter composition elements, considered as non-exhaust road traffic emissions, including copper (Cu), iron (Fe), zinc (Zn), nickel (Ni), sulfur (S), and vanadium (V). These studies exclusively originated from the Dutch PIAMA cohort [48,75]. One study assessed oxidative potential, which is a measure of the inherent capacity of particulate matter to oxidise target molecules, [31], and no studies assessed ultra-fine particles.

### 3.4. Exposure Assessment Place and Time (Windows)

Table S1 in the supplementary material is a summary of where and when the exposure to TRAP was assessed in each included study and whether any validation was undertaken. The assignment of TRAP exposures was almost exclusively based on the residential address of the participating children. Only a few studies considered the impact of moving residence on TRAP exposure levels and undertook additional or sensitivity analyses for movers/non-movers or assigned the exposure at multiple addresses based on the residential history. There were a few studies which assigned the exposure based on school locations instead of residence. Shima and Adachi [79] and Shima et al. [81] used routine measurements from fixed-site stations near school addresses to represent TRAP exposures in Japan, whilst Deng, Lu, Norbäck, Bornehag, Zhang, Liu, Yuan and Sundell [70] and Deng, Lu, Ou, Chen and Yuan [86] used routine measurements from fixed-site stations near children’s kindergartens to represent TRAP exposures in China.

The exposure assignment was generally static; i.e., not taking children’s mobility into account. In many cases, this could be argued as reasonable as participants were in their infancy or early life (birth–3 years old), and residential exposure is then thought to be most relevant. Only 10 studies, mostly recent, considered children’s mobility in the exposure assessment and assigned time-weighted exposures at day cares and/or schools [33,34,38,40,42,50,83,84], and other locations where the child spends significant time [46,76], alongside residence. These studies were conducted at ages when exposure at the residential address becomes less relevant due to children’s increased mobility.

In terms of the exposure time window investigated, studies differed, but birth year was the most explored time window (Table S1). Very few studies investigated alternative exposure windows such as different years of life, longer duration, cumulative or life-time exposure.

### 3.5. Exposure Assessment Validation

Studies using LUR or dispersion modelling validated their modelled exposure estimates against measured concentration using different methods including leave-one-out cross validation procedure (mainly for LUR models) and independent cross validation against fixed-site monitoring stations measurements (mainly for dispersion models). Generally, the validation of the LUR model estimates were not conducted using a separate test validation dataset which significantly limits the comprehensiveness of the validation. No study reported validation against personal exposures.

### 3.6. Risk Estimates by Exposure Assessment Model

Studies using different TRAP surrogates were the least consistent to show an increased asthma risk associated with TRAP. Studies using dispersion model were more consistent in showing associations. For example, out of 8 studies using dispersion models, 5 showed positive and statistically significant risk estimates. Studies using traffic-related air pollutants concentrations at fixed-site monitoring stations, and studies using LUR modelling generally showed an increased asthma risk associated with TRAP. For example, out of 22 studies using LUR models, 17 showed positive and statistically significant risk estimates. The one study that measured NO_2_ exposure at the individual residential level also showed statistically significant associations between the exposure and asthma [74]; so did the one study that used remote sensing [85]. Some of the same studies which found no association between roadway proximity and asthma, found increased risks when employing more refined exposure models such as LUR model estimates [36,37,38,42,45,78].

## 4. Discussion

We found 42 studies that examined the association between TRAP and the subsequent onset of childhood asthma defined as incidence or lifetime prevalence. Exposures metrics differed in terms of their spatial and temporal resolution and their specificity to traffic. LUR modelling was the most commonly used exposure assessment method and NO_2_ was the most commonly studied pollutant. Most studies estimated TRAP exposures at the residential address and only a few considered the mobility of the children and/or their residential address changes. Most studies estimated the TRAP exposures at the first year of life (birth year) and only a few studies assessed the effects of cumulative exposures and/or exposures at different time-windows. Validation was undertaken for LUR and dispersion models estimates only and no study has validated exposure estimates against personal monitored exposures. Although our previous meta-analysis found positive and statistically significant associations for various TRAP exposures (black carbon, NO_2_, PM_2.5_, PM_10_) with the onset asthma [3], further refinement of the exposure assessments may improve the exposure-response functions and shed light on associations with other under-investigated pollutants.

### 4.1. Putative Agents

The prominent focus on NO_2_ in the literature is probably related to the wide availability of this pollutant measure, the ease and relatively low cost to measure it and its relative specificity to road traffic [30]. The focus on NO_2_ in air quality guidelines, plans and mitigation strategies in the EU, and beyond, is perhaps reinforcing the study of this pollutant. Fewer studies measured or modelled PM_2.5_ or particulate components, even though it is more widely implicated in the health effects of air pollution [88]. The cost of measuring and/or modelling PM tends to be higher. The literature, however, suggests that there has been a recent move from studying standard air pollutants to studying other agents, most notably including black and elemental carbon, two agents that are considered as TRAP signatures, but also PM composition elements and other properties such as oxidative potential [31,48,75]. As it stands, there were no studies investigating the impacts of long-term exposure to ultra-fine particles on asthma but there are studies under way to measure ultra-fine particles [89]. The work on PM composition is particularly relevant with the expected wide-spread introduction of electric vehicles and the associated likely reductions of exhaust emissions and increase in non-exhaust emissions [90]. PM composition research could potentially lead to further insight on the putative agents and source of pollutants. For example, Gehring, Beelen, Eeftens, Hoek, de Hoogh, de Jongste, Keuken, Koppelman, Meliefste and Oldenwening [48] suggested that iron, copper, and zinc in PM, reflecting poorly regulated non-exhaust traffic emissions, may increase the risk of asthma and allergy in Dutch schoolchildren. A birth cohort study using oxidative potential measures, particularly using the dithiothreitol assay, found that asthma and other respiratory health outcomes were more strongly related to oxidative potential when compared to PM_2.5_, suggesting that this exposure metric may be closer to the underlying mechanisms [31]. These different measures are rarely studied and should be further explored in future research, principally in locations where ratios between oxidative potential and other TRAP markers such as NO_2_ differ; to determine with more confidence which metric predicts respiratory health better.

### 4.2. Exposure Assessment Methods

Many studies have used LUR modelling to estimate TRAP exposures, partly because of its relatively low costs, ease of implementation and possibility to consider traffic determinants of exposure such as the road network and traffic density. LUR models also tend to provide a good spatial coverage and resolution for TRAP exposure. The LUR method is an empirical method and uses least squares regression to combine measured data with geographic information system (GIS)-based predictor data reflecting pollutant sources, to build a prediction model applicable to non-measured locations, e.g., residential addresses of cohort members. An advantage of LUR models is that they are stable over time [91,92,93]. However, their validation, most commonly undertaken using leave-one-out cross validation procedure, is incomplete. Relatively few studies used air dispersion models which are based on more detailed knowledge of the physical, chemical, and fluid dynamical processes in the atmosphere. Air dispersion models use information on emissions, source characteristics, chemical and physical properties of the pollutants, topography, and meteorology to model the transport and transformation of gaseous or particulate pollutants through the atmosphere to predict air pollutant concentrations. They allow for a finer temporal and spatial resolution of TRAP exposure and specific source apportionment (beyond TRAP) which is valuable when recommending specific policy interventions targeted at specific sources. Yet, their main drawback is related to the quality of the input data; especially the vehicle emission factors which are highly uncertain [94]. Amongst the encountered exposure methods, these two methods are favorable in terms of their spatial and temporal resolution and their specificity to traffic (Table 2). The preferred method for exposure assessment is not so obvious and depends on available resources, the quality of the input data, expertise, place of study and transferability considerations. For example, de Hoogh, et al. [95] found that the median Pearson R (range) correlation coefficients between LUR and air dispersion model estimates for the annual average concentrations of NO_2_, PM_10_ and PM_2.5_ were 0.75 (0.19–0.89), 0.39 (0.23–0.66) and 0.29 (0.22–0.81) for 112,971 (13 study areas), 69,591 (7) and 28,519 (4) addresses respectively, suggesting a much better agreement for NO_2_ than for PM, probably because the main source for NO_2_ is traffic and PM has other sources. The median Pearson R correlation coefficients (range) between air dispersion model estimates and measurements were 0.74 (0.09–0.86) for NO_2_; 0.58 (0.36–0.88) for PM_10_ and 0.58 (0.39–0.66) for PM_2.5_. Wang et al. [96] compared both methods in a study of children’s lung function and found that exposure estimates from LUR and dispersion models correlated very well for PM_2.5_, NO_2_, and black carbon, but not for PM_10_. Health effect estimates did not depend on the type of model used in their population of Dutch children. Yet, with a very limited number of comparison studies, the extent to which estimates of air pollution effects are affected by the choice of exposure model remains unclear. A combination of the LUR and dispersion models may further improve the exposure assessment estimates, possibly accounting for some of the imperfections in the emission databases [97].

Compared to estimates from routine monitoring stations LUR and air dispersion model have the advantage that they provide a better spatial resolution, but also require more effort and are costlier. The better spatial resolution may be quite important when the study area is small and clear exposure differences can be observed by detailed exposure assessment. At least, all three methods provide some level of pollutants which may be important for policy reasons, while surrogate measures like distance from roads do not. A relatively new method, remote sensing [85] has the advantage that air pollution estimates can be obtained where there are no or fewer monitors or less resources and expertise is available i.e., medium- and low-income countries, but still needs some further refinement in terms of spatial resolution and the number of pollutants for which good estimation methods are available.

We attempted to evaluate the effects of the exposure assessment method on the health risk estimates observed in the included studies, for example with meta-regression, but the number of studies available are still too small to conduct such analyses. Even for NO_2_ exposure, there were only 20 studies entering the meta-analysis, 12 of which used LUR models and 1 used dispersion modelling. The meta-analyses though suggested considerable heterogeneity, especially in the case of NO_2_ where most studies where available, and part of this heterogeneity could be caused by different exposure assessment methods. Given the rapid increase in the number of studies in this field, it may become possible to conduct such analysis in the near future.

### 4.3. Exposure Assessment Places and Time Windows

Only a small number of studies considered children’s mobility at ages when exposure at the residential address becomes less relevant and assigned time-weighted TRAP exposures at day care centres and schools and other locations where the child spends significant time alongside residence. Children may spend only around 50%–60% of their time at home, and the rest elsewhere e.g., at school [98]. TRAP exposure levels such as black carbon can be considerably higher when commuting compared to being at home [98], and therefore residential estimates may underestimate the true exposure and bias the exposure-response functions. New tracking technology and portable sensors have now made it possible obtain information on TRAP exposure levels over the day, even though it requires considerable effort and may only feasible for smaller samples [98]. New approaches such as indicating the home and school address and commuting route in geographical information system packages and overlaying this with time adjusted air pollution maps may provide estimates for larger study samples and can be an area of further inquiry. Considering the significant amount of time spent indoors, it may also be beneficial to investigate indoor air pollution exposures and the impact of specifically incorporating these on the exposure–response functions. Currently, all available exposure models, except personal monitors (which have not been used in any of the included studies), estimate outdoor air pollution only and use this as a surrogate for the indoor levels without taking into account indoor-outdoor penetration factors. However, outdoor and indoor TRAP are correlated as there is considerable penetration of outdoor sources to indoor environments. These correlations are may be one rapid and practical method to assign indoor exposures. One study which characterized the indoor–outdoor relationship of PM_2.5_ in Beijing found that there is a strong correlation between indoor and outdoor PM_2.5_ mass concentrations, and that the ambient data explained ≥ 84% variance of the indoor data [99]. Another study similarly showed that PM_2.5_ levels in an Australian primary school were mainly affected by the outdoor PM_2.5_ (r = 0.68, *p* < 0.01) [100]. Another study in Germany found that over 75% of the daily indoor PM_2.5_ and black smoke variation could be explained by daily outdoor variation for those pollutants [101].

Further, investigating different exposure time windows may highlight other relevant exposure windows beyond the birth year and early-life that are commonly studied. The differences between effects of early exposure versus later exposures or exposures with greater duration is yet unclear and is difficult to detangle due to the limited number of studies investigating different time windows. Some authors have suggested that exposures of longer duration at elevated TRAP levels may be necessary to generate pathophysiological changes leading to asthma development and therefore may be behind the observed effects [46].

### 4.4. Outlook and Recommendations

Novel approaches to exposure assessment are underway including the use of OMICS technologies that measure biological molecules and/or activity in the body (e.g., transcriptomics, proteomics, metabolomics or methylation) to identify fingerprints of air pollution [89,102]. Although still in their infancy, such approaches may provide a good way of characterising air pollution exposures inside the body and on existing biological samples (that have been stored for a while). Furthermore, they may provide further insight in the underlying mechanisms by which air pollution cause health effects in children and others.

Although there appear to be statistical significant associations between TRAP and the development of childhood asthma, there is a further need to improve the exposure estimates, and therefore improve the exposure–response functions and the consistency of the study findings. This is important for example when these exposure–response functions are used for burden of disease and health impact assessment studies, and for better understanding the underlying mechanisms of TRAP and childhood asthma and the potential differential pollutant effects and drivers of heterogeneity. Over the past few years, there has been an epidemic increase in the number of studies in the field, and there are likely to be more studies over the next few years given the importance of the topic. Improvements in exposure assessments, as we discuss in this paper, may well increase the scientific value of these new studies. More refined exposure models are needed, and will arguably produce the most robust associations when investigating the potential health effects of TRAP. Furthermore, we also emphasize the need to incorporate mobility patterns in the exposure estimates and to undertake personal exposure monitoring to cross validate modelling estimates.

## 5. Conclusions

Although our previous meta-analysis found statistically significant associations for various TRAP exposures and childhood asthma, further refinement of the exposure assessment may improve the risk estimates and shed light on critical exposure time windows, putative agents, underlying mechanisms and drivers of heterogeneity.

## Figures and Tables

**Table 1:** Main characteristics of the included studies.

Study Reference	Setting	Study Design	Age Group (Years)	Participants Included in the Analysis	Exposure Assessment	Pollutant(s)
Brauer, Hoek, Van Vliet, Meliefste, Fischer, Wijga, Koopman, Neijens, Gerritsen and Kerkhof [68]	The Netherlands, north, west and center communities	Birth cohort (PIAMA)	Birth–2	2989	LUR modelling	BC, NO_2_, PM_2.5_
Brauer, Hoek, Smit, De Jongste, Gerritsen, Postma, Kerkhof and Brunekreef [67]	The Netherlands, north, west and center communities	Follow-up on Brauer et al. (2002)	Birth–4	2826	LUR modelling	BC, NO_2_, PM_2.5_
Brunst, Ryan, Brokamp, Bernstein, Reponen, Lockey, Khurana Hershey, Levin, Grinshpun and LeMasters [46]	USA, Cincinnati	Birth cohort (CCAAPS)	Birth–7	589	LUR modelling	EC
Carlsten, Dybuncio, Becker, Chan-Yeung and Brauer [69]	Canada, Vancouver	Birth cohort (CAPPS)	Birth–7	184	LUR modelling	BC, NO, NO_2_, PM_2.5_
Clark, Demers, Karr, Koehoorn, Lencar, Tamburic and Brauer [37]	Canada, Southwestern British Columbia	Case-control nested in British Columbia birth cohort	Birth–4	37,401	LUR modelling, monitoring data at closest three monitors weighted by inverse distance to child’s residence, proximity to highways/major roads	BC, CO, NO, NO_2_, PM_10_, PM_2.5_
Fuertes, Standl, Cyrys, Berdel, von Berg, Bauer, Krämer, Sugiri, Lehmann and Koletzko [72]	Germany	2 birth cohorts (GINIplus and LISAplus)	3–10	4585	LUR modelling	BC, NO_2_, PM_2.5_
Gehring, Cyrys, Sedlmeir, Brunekreef, Bellander, Fischer, Bauer, Reinhardt, Wichmann and Heinrich [73]	Germany, Munich	2 birth cohorts (GINI and LISA)	Birth–2	1756	LUR modelling	BC, NO_2_, PM_2.5_
Gehring, Wijga, Brauer, Fischer, de Jongste, Kerkhof, Oldenwening, Smit and Brunekreef [39]	The Netherlands, north, west and center communities	Follow-up on Brauer et al. (2007)	Birth–8	3143	LUR modelling	BC, NO_2_, PM_2.5_
Gehring, Beelen, Eeftens, Hoek, de Hoogh, de Jongste, Keuken, Koppelman, Meliefste and Oldenwening [48]	The Netherlands, north, west and center communities	Follow-up on Gehring et al. (2010)	Birth–12	3702	LUR modelling	BC, NO_2_, PM_2.5_, PM_10_, PM_coarse_ and PM composition elements: copper (Cu), iron (Fe), zinc (Zn), nickel (Ni), sulfur (S), vanadium (V)
Gehring, Wijga, Hoek, Bellander, Berdel, Brüske, Fuertes, Gruzieva, Heinrich and Hoffmann [47]	Sweden, Germany, The Netherlands	Pooled data from four birth cohorts: BAMSE; GINIplus; LISAplus and PIAMA	Birth–16	14,126	LUR modelling	BC, NO_2_, PM_2.5_, PM_10_, PM_coarse_
Gruzieva, Bergström, Hulchiy, Kull, Lind, Melén, Moskalenko, Pershagen and Bellander [40]	Sweden, Stockholm	Birth cohort (BAMSE)	Birth–12	3633	Dispersion modelling (Airviro, street canyon contribution for 160 houses)	NO_x_, PM_10_
Jerrett, Shankardass, Berhane, Gauderman, Künzli, Avol, Gilliland, Lurmann, Molitor and Molitor [74]	USA, 11 southern Californian communities	Cohort (CHS)	10–18	209	NO_2_ Palmes tubes monitoring for 2 weeks in 2 seasons at the child’s residence	NO_2_
Kerkhof, Postma, Brunekreef, Reijmerink, Wijga, De Jongste, Gehring and Koppelman [75]	The Netherlands, north, west and center communities	Birth cohort (PIAMA)	Birth–8	916	LUR modelling	BC, NO_2_, PM_2.5_
Krämer, Sugiri, Ranft, Krutmann, von Berg, Berdel, Behrendt, Kuhlbusch, Hochadel and Wichmann [36]	Germany, Wesel	2 birth cohorts (GINIplus and LISAplus)	4–6	2059	LUR modelling, distance to next major road traversed by more than 10,000 cars/day	BC, NO_2_
LeMasters, Levin, Bernstein, Lockey, Lockey, Burkle, Khurana Hershey, Brunst and Ryan [76]	USA, Cincinnati	Birth cohort (CCAAPS)	Birth–7	575	LUR modelling	EC
Lindgren, Stroh, Björk and Jakobsson [41]	Sweden, Scania	Birth cohort	Birth–6	6007	Dispersion modelling (AERMOD), traffic intensity on road with heaviest traffic within 100 m around residence	NO_x_
MacIntyre, Brauer, Melén, Bauer, Bauer, Berdel, Bergström, Brunekreef, Chan-Yeung, Klümper, Fuertes, Gehring, Gref, Heinrich, Herbarth, Kerkhof, Koppelman, Kozyrskyj, Pershagen, Postma, Thiering, Tiesler, Carlsten and Group [52]	Sweden, Canada, Germany, The Netherlands	Pooled data from 6 birth cohorts: BAMSE; CAPPS; GINI; LISA; PIAMA; SAGE	Birth–8	5115	LUR modelling, dispersion modelling for BAMSE only	NO_2_ (sensitivity analyses for BC and PM_2.5_)
McConnell, Islam, Shankardass, Jerrett, Lurmann, Gilliland and Gauderman [42]	USA, 13 southern Californian communities	Cohort (CHS)	Kindergarten/first grade–fourth grade	2497	Dispersion modelling for NOx (CALINE 4), monitoring data for NO_2_, PM_2.5_, PM_10_, distance to nearest freeway or other highways or arterial roads, traffic density within 150 m around residence and school	NO_x_, NO_2_, PM_2.5_, PM_10_
Mölter, Agius, de Vocht, Lindley, Gerrard, Custovic and Simpson [50]	England, Greater Manchester	Birth cohort (MAAS)	Birth–11	1108	Microenvironmental exposure model (LUR modelling for outdoor and INDAIR for indoor environments, indoor to outdoor ratios: journey to school and school)	NO_2_, PM_10_
Mölter, Simpson, Berdel, Brunekreef, Custovic, Cyrys, de Jongste, de Vocht, Fuertes and Gehring [51]	ESCAPE multi-center analysis, England, Sweden, Germany, The Netherlands	Pooled data from 5 birth cohorts: MAAS, BAMSE, PIAMA, GINI, LISA (South and North)	Birth–10	10,377	LUR modelling, traffic intensity on the nearest street, traffic intensity on major roads within a 100-m radius	BC, NO_2_, NO_x_, PM_2.5_, PM_10_, PM_coarse_
Morgenstern, Zutavern, Cyrys, Brockow, Gehring, Koletzko, Bauer, Reinhardt, Wichmann and Heinrich [58]	Germany, Munich Metropolitan area	2 birth cohorts (GINI and LISA)—extension on Gehring et al. (2002)	Birth–2	3577	LUR modelling, living close to major road	BC, NO_2_, PM_2.5_
Morgenstern, Zutavern, Cyrys, Brockow, Koletzko, Kramer, Behrendt, Herbarth, von Berg and Bauer [77]	Germany, Munich	2 birth cohorts (GINI and LISA)	4–6	2436	LUR modelling, minimum distance to next motorway, federal or state road	BC, NO_2_, PM_2.5_
Oftedal, Nystad, Brunekreef and Nafstad [78]	Norway, Oslo	Oslo birth cohort and sample from simultaneous cross-sectional study	Birth–10	2329	Dispersion modelling (EPISODE), distance to main transport routes with any form of motor transport	NO_2_
Patel, Quinn, Jung, Hoepner, Diaz, Perzanowski, Rundle, Kinney, Perera and Miller [44]	USA, New York	Birth cohort (CCCEH)	Birth–5	593	Proximity to roadways, roadway density, truck route density, four-way street intersection density, number of bus stops, percentage of building area designated for commercial use	NA
Rancière [34]	Paris, France	Birth cohort (PARIS)	Birth–4	2015	Dispersion modelling	NO_x_
Ranzi, Porta, Badaloni, Cesaroni, Lauriola, Davoli and Forastiere [45]	Italy, Rome	Birth cohort (GASPII)	Birth–7	672	LUR modelling, proximity to high traffic roads	NO_2_
Shima and Adachi [79]	Japan, 7 Chiba Prefecture communities	Cohort	9/10–12/13	842	Monitoring data	NO_2_
Shima, Nitta, Ando and Adachi [81]	Japan, 8 Chiba Prefecture communities	Cohort	6–12	1910	Monitoring data	NO_2_, PM_10_
Shima, Nitta and Adachi [80]	Japan, 8 Chiba Prefecture communities	Cohort	6/9–10/13	1858	Distance to trunk roads	NA
Tétreault, Doucet, Gamache, Fournier, Brand, Kosatsky and Smargiassi [85]	Canada, Québec	Birth cohort	Birth–12	1,133,938	LUR modelling for NO_2_, satellite imagery for PM_2.5_	NO_2_, PM_2.5_
Wang, Tung, Tang and Zhao [82]	Taiwan, 11 communities in Taipei	Cohort (CEAS)	Birth–kindergarten (average age 5.5 ± 1.1)	2661	Monitoring data	CO, NO_2_, PM_2.5_, PM_10_
Yamazaki, Shima, Nakadate, Ohara, Omori, Ono, Sato and Nitta [83]	Japan, 57 elementary schools	Cohort (SORA)	6–9	10,069	Dispersion modelling for outdoor and indoor concentrations, living near heavily trafficked roads	EC, NO_x_
Yang, Janssen, Brunekreef, Cassee, Hoek and Gehring [31]	The Netherlands, north, west and center communities	Birth cohort (PIAMA)	Birth-14	3701	LUR modelling	Oxidative Potential, BC, NO_2_, PM_2.5_, copper (Cu), iron (Fe), zinc (Zn), nickel (Ni), sulfur (S), vanadium (V)
Dell, Jerrett, Beckerman, Brook, Foty, Gilbert, Marshall, Miller, To and Walter [38]	Canada, Toronto	Case-control	5–9	1497	LUR modelling, monitoring data weighted by inverse distance to child’s residence, distance to highways/major roadways	NO_2_
English, Neutra, Scalf, Sullivan, Waller and Zhu [71]	USA, San Diego	Case-control	≤14	8280	Average daily traffic on streets within a 168-m buffer around residence	NA
Hasunuma, Sato, Iwata, Kohno, Nitta, Odajima, Ohara, Omori, Ono and Yamazaki [33]	Japan, 9 cities and wards	Case-control (nested in SORA)	1.5–3	416	Dispersion modelling including indoor concentration assuming an infiltration rate from outdoor concentration, distance from heavily trafficked roads	EC, NO_x_
[43]	USA, Chicago, Bronx, Houston, San Francisco, Puerto Rico	2 case-controls (GALA II and SAGE II)	8–21	3015	Monitoring data at closest four monitors weighted by inverse distance squared to child’s residence	NO_2_, PM_2.5_, PM_10_
Zmirou, Gauvin, Pin, Momas, Sahraoui, Just, Le Moullec, Bremont, Cassadou and Reungoat [84]	France, Paris, Nice, Toulouse, Clermont-Ferrand, Grenoble	Case-control (VESTA)	4–14	390	Traffic density within 300 m to road distance ratio	NA
Deng, Lu, Norbäck, Bornehag, Zhang, Liu, Yuan and Sundell [70]	China, Changsha	Cross-sectional (CCHH)	3–6	2490	Monitoring data weighted by inverse distance to child’s kindergarten	NO_2_, PM_10_ (as a mixture surrogate)
Deng, Lu, Ou, Chen and Yuan [86]	China, Changsha	Cross-sectional (CCHH)	3–6	2598	Monitoring data weighted by inverse distance to child’s kindergarten	NO_2_, PM_10_ (as a mixture surrogate)
[32]	Korea, 45 elementary schools	Cross-sectional	6–7	1828	Monitoring data	CO, NO_2_, PM_10_
Liu, Huang, Hu, Fu, Zou, Sun, Shen, Wang, Cai and Pan [87]	China, Shanghai	Cross-sectional (CCHH)	4–6	3358	Monitoring data	NO_2_, PM_10_

Abbreviations: BAMSE, Barn (children), Allergy, Milieu, Stockholm, an Epidemiology project; BC: black carbon; CAPPS, The Canadian Asthma Primary Prevention Study; CCAAPS, The Cincinnati Childhood Allergy and Air Pollution Study; CCCEH, Columbia Center for Children’s Environmental Health birth cohort study; CCHH, China-Children-Homes-Health study; CEAS, Childhood Environment and Allergic Diseases Study; CHS, The Children’s Health Study; EC, elemental carbon; ESCAPE, The European Study of Cohorts for Air Pollution Effects; GALA II, The Genes–environments and Admixture in Latino Americans; GASPII, The Gene and Environment Prospective Study in Italy; GINIplus, German Infant study on the influence of Nutrition Intervention plus air pollution and genetics on allergy development; ICD, International Classification of Diseases; LISAplus, Life style Immune System Allergy plus air pollution and genetics; LUR, land-use regression; MAAS, The Manchester Asthma and Allergy Study; Medi-Cal, California Medical Assistance Program; NA, not applicable; NO, nitrogen oxide; PM: particulate matter; SAGE II, The Study of African Americans, Asthma, Genes and Environments; SAGE, The Study of Asthma, Genes and the Environment; SORA, Study on Respiratory Disease and Automobile Exhaust; VESTA, Five (V) Epidemiological Studies on Transport and Asthma; y.o., years old.

**Table 2:** Pros and cons of exposure assessment methods used in the systematic review literature. TRAP: traffic-related air pollution.

Exposure Model	Resolution		Specificity to Traffic	Pros	Cons
	Spatial	Temporal			
TRAP surrogates main e.g., proximity to “major roads” or “freeways”	-	--	+	Intuitive, simple and cost effective, more insightful when complemented with vehicle counts and composition, low need for updated data.	Assumes a road of a certain type or size corresponds to a certain amount of traffic, sometime uses self-reported traffic intensity (collected via questionnaires) which can be subjective, assumes all pollutants disperse similarly (limited directional dependence), cannot consider street canyon effects, generally does not consider compounded effects of proximity to multiple roads, disregards exposure variability due to mobility/individual activity.
Air pollutants measurements from fixed-site monitoring stations	--	++	--	High and continuous temporal resolution, actual measurements rather than predictions, cost-effective, can provide large sample sizes, medium need for updated data.	Not present at all locations, locations usually based on regulatory (not scientific) purposes, cannot consider street canyon effects (unless located in a street canyon), conceals persons’ differences because of a mismatch between data used to estimate exposure and actual subjects’ locations, potential for significant amounts of missing data in practice, quality of the data depends on quality of data ratification and verification, disregards exposure variability due to mobility/individual activity.
Air pollutant measurements from residential (stationary) samplers	++	-	-	Provides individualized data, captures spatial variability in exposure between study subjects, actual measurements rather than predictions, cost effective for select pollutants (e.g., NO_2_), medium need for updated data.	Only practical/feasible in small timeframes and populations, logistic and costs concerns, not available or cost prohibitive (e.g., ultra-fine particles) for all pollutants of concern, disregards exposure variability due to mobility/individual activity.
Remote sensing	+	-	--	Can provide estimate for large areas, can provide estimate areas where measurements or models are not available (e.g., low income countries), relatively standardized method across regions, medium need for updated data.	Availability depends on satellite presence (i.e., time resolution is limited), crude spatial resolution (10 * 10 km), only available for select pollutants, challenging to assess errors in estimates, cannot consider street canyon effects, disregards exposure variability due to mobility/individual activity.
Land-use regression models	+	--	+	Assume independence between sampled locations, good agreement between measured and predicted averages of NO_2_, less with PM, modelling based on measurements and information around measurement points, relatively easy to collate input data, practical, low costs, medium need for updated data.	Only reflect the predictors used in the model, subject to varying uncertainties amongst different pollutants, the true contribution of traffic to the regression is not always known or reported, difficult to take into account street canyon effects; meteorology and atmospheric chemistry, the quality of the data representing “meaningful” predictors may be an issue and will affect the overall accuracy of the model, the model’s outputs are sensitive to the locations and density of the sampling sites, generally disregards exposure variability due to mobility/individual activity.
Air dispersion models	++	++	++	Continuous exposure metric, traffic-specific i.e., based on traffic flows and flow mix, traffic emissions, meteorology and atmospheric chemistry, covers relatively large areas, can assess episodic short-term and long-term exposures, can consider street canyon effects through optional built-in street canyon model, considers compounded effects of proximity to multiple roads, medium need for updated data.	Severe data demands, resource intensive, at the mercy of the emission factors inputted in the model (subject to high uncertainty), meteorology at the exposure scale is influenced by complex physical features including traffic turbulence which is difficult to consider, overestimates pollution levels during periods of calm wind, generally disregards exposure variability due to mobility/individual activity.

Ratings: +: good; ++: very good; -: potentially inadequate; --: highly inadequate.

## Supplementary Materials

The following are available online at www.mdpi.com/1660-4601/14/3/312/s1, Table S1: Exposure assessment place, time and validation in the Included Studies.

## References

[1] Perez L., Declercq C., Iñiguez C., Aguilera I., Badaloni C., Ballester F., Bouland C., Chanel O., Cirarda F., Forastiere F. (2013). Chronic burden of near-roadway traffic pollution in 10 european cities (APHEKOM network). Eur. Respir. J..

[2] Milligan K.L., Matsui E., Sharma H. (2016). Asthma in urban children: Epidemiology, environmental risk factors, and the public health domain. Curr. Allergy Asthma Rep..

[3] Khreis H., Kelly C., Tate J., Parslow R., Lucas K., Nieuwenhuijsen M. (2017). Exposure to traffic-related air pollution and risk of development of childhood asthma: A systematic review and meta-analysis. Environ. Int..

[4] Committee on the Medical Effects of Air Pollutants (COMEAP) (2014). Asthma and Air Pollution.

[5] London S.J. (2007). Gene–air pollution interactions in asthma. Proc. Am. Thorac. Soc..

[6] Li N., Sioutas C., Cho A., Schmitz D., Misra C., Sempf J., Wang M., Oberley T., Froines J., Nel A. (2003). Ultrafine particulate pollutants induce oxidative stress and mitochondrial damage. Environ. Health Perspect..

[7] Kelly F.J. (2003). Oxidative stress: Its role in air pollution and adverse health effects. Occup. Environ. Med..

[8] Sundvor I., Castell Balaguer N., Viana M., Querol X., Reche C., Amato F., Mellios G., Guerreiro C. Road Traffic’s Contribution to Air Quality in European Cities, ETC/ACM Technical Paper 2012/14. http://acm.eionet.europa.eu/reports/docs/ETCACM_TP_2012_14_traffic_contribution_city_aq.pdf.

[9] Victoria Transport Policy Institute (2013). Transportation Cost and Benefit Analysis II—Air Pollution Costs.

[10] Vardoulakis S., Fisher B.E., Pericleous K., Gonzalez-Flesca N. (2003). Modelling air quality in street canyons: A review. Atmos. Environ..

[11] Jerrett M., Arain A., Kanaroglou P., Beckerman B., Potoglou D., Sahsuvaroglu T., Morrison J., Giovis C. (2005). A review and evaluation of intraurban air pollution exposure models. J. Expo. Sci. Environ. Epidemiol..

[12] Beelen R., Voogt M., Duyzer J., Zandveld P., Hoek G. (2010). Comparison of the performances of land use regression modelling and dispersion modelling in estimating small-scale variations in long-term air pollution concentrations in a Dutch urban area. Atmos. Environ..

[13] Sellier Y., Galineau J., Hulin A., Caini F., Marquis N., Navel V., Bottagisi S., Giorgis-Allemand L., Jacquier C., Slama R. (2014). Health effects of ambient air pollution: Do different methods for estimating exposure lead to different results?. Environ. Int..

[14] Gulliver J., de Hoogh K., Fecht D., Vienneau D., Briggs D. (2011). Comparative assessment of gis-based methods and metrics for estimating long-term exposures to air pollution. Atmos. Environ..

[15] Anderson H.R., Favarato G., Atkinson R.W. (2013). Long-term exposure to air pollution and the incidence of asthma: Meta-analysis of cohort studies. Air Qual. Atmos. Health.

[16] Khreis H., De Hoogh K., Mueller N., Rojas-Rueda D., Tate J., Lucas K., Parslow R., Nieuwenhuijsen M.J. (2017). Traffic-related air pollution and the local chronic burden of childhood asthma in Bradford, UK.

[17] Health Effects Institute (2010). Traffic-Related Air Pollution: A Critical Review of the Literature on Emissions, Exposure, and Health Effects.

[18] Akers J., Aguiar-Ibáñez R., Baba-Akbari Sari A. (2009). CRD’S Guidance for Undertaking Reviews in Health Care.

[19] Khreis H., Kelly C., Tate J., Parslow R. Exposure to Traffic-Related Air Pollution and the Development of Childhood Asthma. http://www.crd.york.ac.uk/PROSPERO/display_record.asp?ID=CRD42014015448.

[20] Vitnerova N., Horstman D., Hnizdova E. (1999). Prevalence priznaku chorob dychaciho traktu u deti skolniho veku Zijicich v oblastech s rozdilnym znecistenim Ovzdusi. Hygiena.

[21] Salameh P., Karaki C., Awada S., Rachidi S., Al Hajje A., Bawab W., Saleh N., Waked M. (2015). Asthme, pollutions intérieure et extérieure: Tude pilote chez des adolescents libanais scolarisés. Rev. Mal. Respir..

[22] Veremchuk L., Cherpack N., Gvozdenko T., Volkova M. (2014). Methodology for the assessment of the impact of the atmospheric air pollution on the formation of the levels of overall morbidity rate of bronchial asthma. Gig. Sanit..

[23] Braback L., Forsberg B. (2009). Does traffic exhaust contribute to the development of asthma and allergic sensitization in children: Findings from recent cohort studies. Environ. Health.

[24] Gasana J., Dillikar D., Mendy A., Forno E., Ramos Vieira E. (2012). Motor vehicle air pollution and asthma in children: A meta-analysis. Environ. Res..

[25] Gowers A.M., Cullinan P., Ayres J.G., Anderson H., Strachan D.P., Holgate S.T., Mills I.C., Maynard R.L. (2012). Does outdoor air pollution induce new cases of asthma? Biological plausibility and evidence: A review. Respirology.

[26] Salam M.T., Islam T., Gilliland F.D. (2008). Recent evidence for adverse effects of residential proximity to traffic sources on asthma. Curr. Opin. Pulm. Med..

[27] Sarnat J.A., Holguin F. (2007). Asthma and air quality. Curr. Opin. Pulm. Med..

[28] Wong G.W.K., Leung T.F. (2005). The effects of air pollution on asthma in children. Clin. Pulm. Med..

[29] Bowatte G., Lodge C., Lowe A.J., Erbas B., Perret J., Abramson M.J., Matheson M., Dharmage S. (2015). The influence of childhood traffic-Elated air pollution exposure on asthma, allergy and sensitization: A systematic review and a meta-Analysis of birth cohort studies. Allergy.

[30] Favarato G., Anderson H.R., Atkinson R., Fuller G., Mills I., Walton H. (2014). Traffic-related pollution and asthma prevalence in children. Quantification of associations with nitrogen dioxide. Air Qual. Atmos. Health.

[31] Yang A., Janssen N.A., Brunekreef B., Cassee F.R., Hoek G., Gehring U. (2016). Children’s respiratory health and oxidative potential of PM_2.5_: The piama birth cohort study. Occup. Environ. Med..

[32] Kim J., Han Y., Seo S.C., Lee J.Y., Choi J., Kim K.H., Woo S.-Y., Kim E.-H., Kwon H.-J., Cheong H.K. (2016). Association of carbon monoxide levels with allergic diseases in children. Allergy Asthma Proc..

[33] Hasunuma H., Sato T., Iwata T., Kohno Y., Nitta H., Odajima H., Ohara T., Omori T., Ono M., Yamazaki S. (2016). Association between traffic-related air pollution and asthma in preschool children in a national japanese nested case–control study. BMJ Open.

[34] Rancière F., Bougas N., Viola M., Momas I. (2016). Early exposure to traffic-related air pollution, respiratory symptoms at 4 years of age, and potential effect modification by parental allergy, stressful family events, and gender: A prospective follow-up study of the paris birth cohort. Environ. Health Perspect..

[35] World Health Organization Maternal, Newborn, Child and Adolescent Health: Adolescent Development. http://www.who.int/maternal_child_adolescent/topics/adolescence/dev/en/.

[36] Krämer U., Sugiri D., Ranft U., Krutmann J., von Berg A., Berdel D., Behrendt H., Kuhlbusch T., Hochadel M., Wichmann H.-E. (2009). Eczema, respiratory allergies, and traffic-related air pollution in birth cohorts from small-town areas. J. Dermatol. Sci..

[37] Clark N.A., Demers P.A., Karr C.J., Koehoorn M., Lencar C., Tamburic L., Brauer M. (2010). Effect of early life exposure to air pollution on development of childhood asthma. Environ. Health Perspect..

[38] Dell S.D., Jerrett M., Beckerman B., Brook J.R., Foty R.G., Gilbert N.L., Marshall L., Miller J.D., To T., Walter S.D. (2014). Presence of other allergic disease modifies the effect of early childhood traffic-related air pollution exposure on asthma prevalence. Environ. Int..

[39] Gehring U., Wijga A.H., Brauer M., Fischer P., de Jongste J.C., Kerkhof M., Oldenwening M., Smit H.A., Brunekreef B. (2010). Traffic-related air pollution and the development of asthma and allergies during the first 8 years of life. Am. J. Respir. Crit. Care Med..

[40] Gruzieva O., Bergström A., Hulchiy O., Kull I., Lind T., Melén E., Moskalenko V., Pershagen G., Bellander T. (2013). Exposure to air pollution from traffic and childhood asthma until 12 years of age. Epidemiology.

[41] Lindgren A., Stroh E., Björk J., Jakobsson K. (2013). Asthma incidence in children growing up close to traffic: A registry-based birth cohort. Environ. Health.

[42] McConnell R., Islam T., Shankardass K., Jerrett M., Lurmann F., Gilliland F., Gauderman J. (2010). Childhood incident asthma and traffic-related air pollution at home and school. Environ. Health Perspect..

[43] Nishimura K.K., Galanter J.M., Roth L.A., Oh S.S., Thakur N., Nguyen E.A., Thyne S., Farber H.J., Serebrisky D., Kumar R. (2013). Early-life air pollution and asthma risk in minority children. The GALA II and SAGE II studies. Am. J. Respir. Crit. Care Med..

[44] Patel M.M., Quinn J.W., Jung K.H., Hoepner L., Diaz D., Perzanowski M., Rundle A., Kinney P.L., Perera F.P., Miller R.L. (2011). Traffic density and stationary sources of air pollution associated with wheeze, asthma, and immunoglobulin e from birth to age 5 years among New York city children. Environ. Res..

[45] Ranzi A., Porta D., Badaloni C., Cesaroni G., Lauriola P., Davoli M., Forastiere F. (2014). Exposure to air pollution and respiratory symptoms during the first 7 years of life in an italian birth cohort. Occup. Environ. Med..

[46] Brunst K.J., Ryan P.H., Brokamp C., Bernstein D., Reponen T., Lockey J., Khurana Hershey G.K., Levin L., Grinshpun S.A., LeMasters G. (2015). Timing and duration of traffic-related air pollution exposure and the risk for childhood wheeze and asthma. Am. J. Respir. Crit. Care Med..

[47] Gehring U., Wijga A.H., Hoek G., Bellander T., Berdel D., Brüske I., Fuertes E., Gruzieva O., Heinrich J., Hoffmann B. (2015). Exposure to air pollution and development of asthma and rhinoconjunctivitis throughout childhood and adolescence: A population-based birth cohort study. Lancet Respir. Med..

[48] Gehring U., Beelen R., Eeftens M., Hoek G., de Hoogh K., de Jongste J.C., Keuken M., Koppelman G.H., Meliefste K., Oldenwening M. (2015). Particulate matter composition and respiratory health: The piama birth cohort study. Epidemiology.

[49] Ryan P.H., LeMasters G.K., Levin L., Burkle J., Biswas P., Hu S., Grinshpun S., Reponen T. (2008). A land-use regression model for estimating microenvironmental diesel exposure given multiple addresses from birth through childhood. Sci. Total Environ..

[50] Mölter A., Agius R., de Vocht F., Lindley S., Gerrard W., Custovic A., Simpson A. (2014). Effects of long-term exposure to PM_10_ and NO_2_ on asthma and wheeze in a prospective birth cohort. J. Epidemiol. Community Health.

[51] Mölter A., Simpson A., Berdel D., Brunekreef B., Custovic A., Cyrys J., de Jongste J., de Vocht F., Fuertes E., Gehring U. (2015). A multicentre study of air pollution exposure and childhood asthma prevalence: The escape project. Eur. Respir. J..

[52] MacIntyre E.A., Brauer M., Melén E., Bauer C.P., Bauer M., Berdel D., Bergström A., Brunekreef B., Chan-Yeung M., Klümper C. (2014). Gstp1 and TNF gene variants and associations between air pollution and incident childhood asthma: The traffic, asthma and genetics (TAG) study. Environ. Health Perspect..

[53] Gauderman W.J., Avol E., Lurmann F., Kuenzli N., Gilliland F., Peters J., McConnell R. (2005). Childhood asthma and exposure to traffic and nitrogen dioxide. Epidemiology.

[54] Gruzieva O., Bellander T., Eneroth K., Kull I., Melén E., Nordling E., van Hage M., Wickman M., Moskalenko V., Hulchiy O. (2012). Traffic-related air pollution and development of allergic sensitization in children during the first 8 years of life. J. Allergy Clin. Immunol..

[55] Henderson S.B., Beckerman B., Jerrett M., Brauer M. (2007). Application of land use regression to estimate long-term concentrations of traffic-related nitrogen oxides and fine particulate matter. Environ. Sci. Technol..

[56] Hochadel M., Heinrich J., Gehring U., Morgenstern V., Kuhlbusch T., Link E., Wichmann H.-E., Krämer U. (2006). Predicting long-term average concentrations of traffic-related air pollutants using gis-based information. Atmos. Environ..

[57] Jerrett M., Arain M., Kanaroglou P., Beckerman B., Crouse D., Gilbert N., Brook J., Finkelstein N., Finkelstein M. (2007). Modeling the intraurban variability of ambient traffic pollution in Toronto, Canada. J. Toxicol. Environ. Health Part A.

[58] Morgenstern V., Zutavern A., Cyrys J., Brockow I., Gehring U., Koletzko S., Bauer C.-P., Reinhardt D., Wichmann H.-E., Heinrich J. (2007). Respiratory health and individual estimated exposure to traffic-related air pollutants in a cohort of young children. Occup. Environ. Med..

[59] Oftedal B., Walker S.-E., Gram F., McInnes H., Nafstad P. (2008). Modelling long-term averages of local ambient air pollution in Oslo, Norway: Evaluation of nitrogen dioxide, PM_10_ and PM_2.5_. Int. J. Environ. Pollut..

[60] Stroh E., Rittner R., Oudin A., Ardö J., Jakobsson K., Björk J., Tinnerberg H. (2012). Measured and modeled personal and environmental NO_2_ exposure. Popul. Health Metr..

[61] Ryan P.H., LeMasters G.K., Biswas P., Levin L., Hu S., Lindsey M., Bernstein D.I., Lockey J., Villareal M., Hershey G.K.K. (2007). A comparison of proximity and land use regression traffic exposure models and wheezing in infants. Environ. Health Perspect..

[62] Beelen R., Hoek G., Vienneau D., Eeftens M., Dimakopoulou K., Pedeli X., Tsai M.-Y., Künzli N., Schikowski T., Marcon A. (2013). Development of NO_2_ and NO*_x_* land use regression models for estimating air pollution exposure in 36 study areas in europe–the escape project. Atmos. Environ..

[63] Eeftens M., Beelen R., de Hoogh K., Bellander T., Cesaroni G., Cirach M., Declercq C., Dedele A., Dons E., de Nazelle A. (2012). Development of land use regression models for PM_2.5_, PM_2.5_ absorbance, PM_10_ and pmcoarse in 20 European study areas; results of the escape project. Environ. Sci. Technol..

[64] Crouse D.L., Goldberg M.S., Ross N.A. (2009). A prediction-based approach to modelling temporal and spatial variability of traffic-related air pollution in Montreal, Canada. Atmos. Environ..

[65] Nordling E., Berglind N., Melén E., Emenius G., Hallberg J., Nyberg F., Pershagen G., Svartengren M., Wickman M., Bellander T. (2008). Traffic-related air pollution and childhood respiratory symptoms, function and allergies. Epidemiology.

[66] Bellander T., Berglind N., Gustavsson P., Jonson T., Nyberg F., Pershagen G., Järup L. (2001). Using geographic information systems to assess individual historical exposure to air pollution from traffic and house heating in Stockholm. Environ. Health Perspect..

[67] Brauer M., Hoek G., Smit H., De Jongste J., Gerritsen J., Postma D.S., Kerkhof M., Brunekreef B. (2007). Air pollution and development of asthma, allergy and infections in a birth cohort. Eur. Respir. J..

[68] Brauer M., Hoek G., Van Vliet P., Meliefste K., Fischer P.H., Wijga A., Koopman L.P., Neijens H.J., Gerritsen J., Kerkhof M. (2002). Air pollution from traffic and the development of respiratory infections and asthmatic and allergic symptoms in children. Am. J. Respir. Crit. Care Med..

[69] Carlsten C., Dybuncio A., Becker A., Chan-Yeung M., Brauer M. (2011). Traffic-related air pollution and incident asthma in a high-risk birth cohort. Occup. Environ. Med..

[70] Deng Q., Lu C., Norbäck D., Bornehag C.-G., Zhang Y., Liu W., Yuan H., Sundell J. (2015). Early life exposure to ambient air pollution and childhood asthma in China. Environ. Res..

[71] English P., Neutra R., Scalf R., Sullivan M., Waller L., Zhu L. (1999). Examining associations between childhood asthma and traffic flow using a geographic information system. Environ. Health Perspect..

[72] Fuertes E., Standl M., Cyrys J., Berdel D., von Berg A., Bauer C.-P., Krämer U., Sugiri D., Lehmann I., Koletzko S. (2013). A longitudinal analysis of associations between traffic-related air pollution with asthma, allergies and sensitization in the GINIplus and LISAplus birth cohorts. PeerJ.

[73] Gehring U., Cyrys J., Sedlmeir G., Brunekreef B., Bellander T., Fischer P., Bauer C., Reinhardt D., Wichmann H., Heinrich J. (2002). Traffic-related air pollution and respiratory health during the first 2 yrs of life. Eur. Respir. J..

[74] Jerrett M., Shankardass K., Berhane K., Gauderman W.J., Künzli N., Avol E., Gilliland F., Lurmann F., Molitor J.N., Molitor J.T. (2008). Traffic-related air pollution and asthma onset in children: A prospective cohort study with individual exposure measurement. Environ. Health Perspect..

[75] Kerkhof M., Postma D., Brunekreef B., Reijmerink N., Wijga A., De Jongste J., Gehring U., Koppelman G. (2010). Toll-like Receptor 2 and 4 genes influence susceptibility to adverse effects of traffic-related air pollution on childhood asthma. Thorax.

[76] LeMasters G., Levin L., Bernstein D.I., Lockey S.D., Lockey J.E., Burkle J., Khurana Hershey G.K., Brunst K., Ryan P.H. (2015). Secondhand smoke and traffic exhaust confer opposing risks for asthma in normal and overweight children. Obesity.

[77] Morgenstern V., Zutavern A., Cyrys J., Brockow I., Koletzko S., Kramer U., Behrendt H., Herbarth O., von Berg A., Bauer C.P. (2008). Atopic diseases, allergic sensitization, and exposure to traffic-related air pollution in children. Am. J. Respir. Crit. Care Med..

[78] Oftedal B., Nystad W., Brunekreef B., Nafstad P. (2009). Long-term traffic-related exposures and asthma onset in schoolchildren in Oslo, Norway. Environ. Health Perspect..

[79] Shima M., Adachi M. (2000). Effect of outdoor and indoor nitrogen dioxide on respiratory symptoms in schoolchildren. Int. J. Epidemiol..

[80] Shima M., Nitta Y., Adachi M. (2003). Traffic-related air pollution and respiratory symptoms in children living along trunk roads in Chiba prefecture, Japan. J. Epidemiol..

[81] Shima M., Nitta Y., Ando M., Adachi M. (2002). Effects of air pollution on the prevalence and incidence of asthma in children. Arch. Environ. Health.

[82] Wang I.-J., Tung T.-H., Tang C.-S., Zhao Z.-H. (2016). Allergens, air pollutants, and childhood allergic diseases. Int. J. Hyg. Environ. Health.

[83] Yamazaki S., Shima M., Nakadate T., Ohara T., Omori T., Ono M., Sato T., Nitta H. (2014). Association between traffic-related air pollution and development of asthma in school children: Cohort study in Japan. J. Expo. Sci. Environ. Epidemiol..

[84] Zmirou D., Gauvin S., Pin I., Momas I., Sahraoui F., Just J., Le Moullec Y., Bremont F., Cassadou S., Reungoat P. (2004). Traffic related air pollution and incidence of childhood asthma: Results of the Vesta case-control study. J. Epidemiol. Community Health.

[85] Tétreault L.-F., Doucet M., Gamache P., Fournier M., Brand A., Kosatsky T., Smargiassi A. (2016). Childhood exposure to ambient air pollutants and the onset of asthma: An administrative cohort study in Québec. Environ. Health Perspect..

[86] Deng Q., Lu C., Ou C., Chen L., Yuan H. (2016). Preconceptional, prenatal and postnatal exposure to outdoor and indoor environmental factors on allergic diseases/symptoms in preschool children. Chemosphere.

[87] Liu W., Huang C., Hu Y., Fu Q., Zou Z., Sun C., Shen L., Wang X., Cai J., Pan J. (2016). Associations of gestational and early life exposures to ambient air pollution with childhood respiratory diseases in shanghai, china: A retrospective cohort study. Environ. Int..

[88] Héroux M.-E., Anderson H.R., Atkinson R., Brunekreef B., Cohen A., Forastiere F., Hurley F., Katsouyanni K., Krewski D., Krzyzanowski M. (2015). Quantifying the health impacts of ambient air pollutants: Recommendations of a who/europe project. Int. J. Public Health.

[89] Vineis P., Chadeau-Hyam M., Gmuender H., Gulliver J., Herceg Z., Kleinjans J., Kogevinas M., Kyrtopoulos S., Nieuwenhuijsen M., Phillips D. (2016). The exposome in practice: Design of the exposomics project. Int. J. Hyg. Environ. Health.

[90] Timmers V.R., Achten P.A. (2016). Non-exhaust pm emissions from electric vehicles. Atmos. Environ..

[91] Gulliver J., de Hoogh K., Hansell A., Vienneau D. (2013). Development and back-extrapolation of NO_2_ land use regression models for historic exposure assessment in Great Britain. Environ. Sci. Technol..

[92] Wang R., Henderson S.B., Sbihi H., Allen R.W., Brauer M. (2013). Temporal stability of land use regression models for traffic-related air pollution. Atmos. Environ..

[93] Cesaroni G., Porta D., Badaloni C., Stafoggia M., Eeftens M., Meliefste K., Forastiere F. (2012). Nitrogen dioxide levels estimated from land use regression models several years apart and association with mortality in a large Cohort study. Environ. Health.

[94] Carslaw D., Beevers S., Westmoreland E., Williams M., Tate J., Murrells T., Stedman J., Li Y., Grice S., Kent A. (2011). Trends in NO_x_ and NO_2_ Emissions and Ambient Measurements in the UK.

[95] De Hoogh K., Korek M., Vienneau D., Keuken M., Kukkonen J., Nieuwenhuijsen M.J., Badaloni C., Beelen R., Bolignano A., Cesaroni G. (2014). Comparing land use regression and dispersion modelling to assess residential exposure to ambient air pollution for epidemiological studies. Environ. Int..

[96] Wang M., Gehring U., Hoek G., Keuken M., Jonkers S., Beelen R., Eeftens M., Postma D.S., Brunekreef B. (2015). Air pollution and lung function in dutch children: A comparison of exposure estimates and associations based on land use regression and dispersion exposure modeling approaches. Environ. Health Perspect..

[97] Korek M., Johansson C., Svensson N., Lind T., Beelen R., Hoek G., Pershagen G., Bellander T. (2016). Can dispersion modeling of air pollution be improved by land-use regression? An example from Stockholm, Sweden. J. Expo. Sci. Environ. Epidemiol..

[98] Nieuwenhuijsen M.J., Donaire-Gonzalez D., Rivas I., De Castro M., Cirach M., Hoek G., Seto E., Jerrett M., Sunyer J. (2015). Variability in and agreement between modeled and personal continuously measured black carbon levels using novel smartphone and sensor technologies. Environ. Sci. Technol..

[99] Huang L., Pu Z., Li M., Sundell J. (2015). Characterizing the indoor-outdoor relationship of fine particulate matter in non-heating season for urban residences in Beijing. PLoS ONE.

[100] Guo H., Morawska L., He C., Zhang Y.L., Ayoko G., Cao M. (2010). Characterization of particle number concentrations and PM_2.5_ in a school: Influence of outdoor air pollution on indoor air. Environ. Sci. Pollut. Res..

[101] Cyrys J., Pitz M., Bischof W., Wichmann H.-E., Heinrich J. (2004). Relationship between indoor and outdoor levels of fine particle mass, particle number concentrations and black smoke under different ventilation conditions. J. Expo. Sci. Environ. Epidemiol..

[102] Vrijheid M., Slama R., Robinson O., Chatzi L., Coen M., van den Hazel P., Thomsen C., Wright J., Athersuch T.J., Avellana N. (2014). The human early-life exposome (HELIX): Project rationale and design. Environ. Health Perspect..

