# Supplementary material for: Traffic-Related Air Pollution and Childhood Asthma: Recent Advances and Remaining Gaps in the Exposure Assessment Methods
Source: Int J Environ Res Public Health. 2017 Mar 17;14(3):312. doi: 10.3390/ijerph14030312 (PMC5369148; doi:10.3390/ijerph14030312)
Supplement: Supplementary file 1 [file ijerph-14-00312-s001.pdf]

Table S1 Exposure assessment place, time and validation in the Included Studies

Exposure assessment method	Study reference	Exposure assessment place	Exposure assessment time	Exposure assessment validation
**TRAP surrogates**	Clark et al. (2010)	Residential address<br>(**considering moving residence**)	Pregnancy and birth year	No validation
	Dell et al. (2014)	Residential address	Birth year and current (year of recruitment)	No validation
	English et al. (1999)	Residential address within buffer region	Current (year of recruitment)	No validation
	Hasunuma et al. (2016)	UAt home. At school for part of the population and considering mobility	At 1.5 years	No validation
	Krämer et al. (2009)	Residential address<br>(**considering moving residence**)	Birth year	No validation
	Lindgren et al. (2013)	Residential address<br>(**additional analysis with children censored when/if moving during time at risk**)	Birth year	No validation
	McConnell et al. (2010)	Residential and school addresses	Year of recruitment	No validation
	Mölter et al. (2014b)	Residential address at birth, 4-5 years and at 8-10 years	Birth year<br>At 4-5 y.o.<br>At 8-10 y.o.	No validation
	Morgenstern et al. (2007)	Residential address	Birth year	No validation

	Morgenstern et al. (2008)	Residential address	Birth year	No validation
	Oftedal et al. (2009)	Residential address	Birth year	No validation
	Patel et al. (2011)	Residential address<br>**(additional analysis for non-movers)**	At 1 y.o.<br>At 2 y.o.<br>At 3 y.o.	No validation
	Ranzi et al. (2014)	Residential address	Birth year	No validation
	Shima et al. (2003)	Residential address	Year of recruitment	No validation
	Yamazaki et al. (2014)	Houses and schools	Unclear	No validation
	Zmirou et al. (2004)	Residential and daycare or school addresses<br>(**considering moving residence**)	Before the age of 3 y.o.<br>Lifelong	No validation
**Fixed-site monitoring stations**	Clark et al. (2010)	Residential address<br>(**considering moving residence**)	Pregnancy and birth year	No validation
	Dell et al. (2014)	Residential address	Birth year, current (year of recruitment) and cumulative	No validation
	Deng et al. (2015)	Kindergarten address<br>(**additional analysis for non-movers**)	Pregnancy and birth year	No validation
	Deng et al. (2016)	Kindergarten address	Pre-conceptional (exposure during 1 year before conception), pre-natal (exposure during the whole pregnancy), and post-natal exposure (exposure from birth to the current)	No validation

	Kim et al. (2016)	Residential address	Birth year and the year preceding the questionnaire survey	No validation
	Liu et al. (2016)	Residential address	Pregnancy year, first year, first two years, first three years, and total years since birth (from birth day to the surveyed day)	No validation
	McConnell et al. (2010)	Residential area/district	The 2003 average ambient concentrations were assigned to all children in the community (exposure at the recruitment)	No validation
	Nishimura et al. (2013)	Residential address<br>(**considering moving residence**)	Birth year and first 3 years of life	No validation
	Shima and Adachi (2000)	School area/district	3 years' average (1991-1993) before/at recruitment (in 1992)	No validation
	Shima et al. (2002)	School area/district<br>(**children had to live at their address 3 years prior to entering the first grade**)	10 years' average (1988-1997)	No validation
	Wang et al. (2016)	Residential address	Long-term cumulative exposure from birth until end of the study	No validation
**Land-use regression models**	Brauer et al. (2002)	Residential address<br>(**additional analysis for non-movers)**	Birth address (long-term exposure)	BC, R^2 = 81%<br>NO_2, R^2 = 85%<br>$PM_{2.5}$, R^2 = 73%
	Brauer et al. (2007)	Residential address	Birth address (long-term exposure)	As above
	Gehring et al. (2010)	Residential address<br>(**additional analysis for non-movers)**	Birth address (long-term exposure)	As above

	Brunst et al. (2015)	Addresses of all locations where the child spent 8 or more hours in an average week (e.g., home, daycare, relative's home, school etc.)	Time-weighted average (birth, birth-1, 1-2, 2-3, 3-4, 6-7, birth-4, 4-6, birth-7 y.o.)	EC, R^2 = 75%
	Carlsten et al. (2010)	Residential address	Birth year	BC, R^2 = 39% - 41%<br>NO, R^2 = 57% - 62%<br>NO_2, R^2 = 56% - 60%<br>$PM_{2.5}$, R^2 = 52%
	Clark et al. (2010)	Residential address<br>(**considering moving residence**)	Pregnancy and birth year	As above
	Dell et al. (2014)	Residential address, daycare and school	Birth year, current (year of recruitment) and cumulative	NO_2, R^2 = 69%
	Fuertes et al. (2013)	Residential address	Birth year, at 6 y.o. and 10 y.o.	BC, R^2 = 91% - 97%<br>NO_2, R^2 = 81% - 89%<br>$PM_{2.5}$, R^2 = 55% - 88%
	Gehring et al. (2002)	Residential address	Birth address (annual average)	BC, R^2 = 67%<br>NO_2, R^2 = 62%<br>$PM_{2.5}$, R^2 = 56%
	Gehring et al. (2015a)	Residential address	"long-term" at the birth and current address	BC, R^2 = 89%<br>NO_2, R^2 = 81%<br>$PM_{2.5}$, R^2 = 61%

				PM_{10}, R^2 = 60%<br>PM_{coarse}, R^2 = 38%<br>Cu $PM_{2.5}$, R^2 = 81%<br>Cu PM_{10}, R^2 = 71%<br>Fe $PM_{2.5}$, R^2 = 73%<br>Fe PM_{10}, R^2 = 70%<br>Ni $PM_{2.5}$, R^2 = 72%<br>Ni PM_{10}, R^2 = 73%<br>S $PM_{2.5}$, R^2 = 27%<br>S PM_{10}, R^2 = 39%<br>V $PM_{2.5}$, R^2 = 63%<br>V PM_{10}, R^2 = 67%<br>Zn $PM_{2.5}$, R^2 = 58%<br>Zn PM_{10}, R^2 = 67%
	Gehring et al. (2015b)	Residential address	Birth and current year (at the different follow-up visits)	BC, R^2 = 81% - 97%<br>NO_2, R^2 = 82% - 89%<br>$PM_{2.5}$, R^2 = 67% - 88%<br>PM_{10}, R^2 = 68% - 83%

				PM_{coarse}, R^2 = 51% - 81%
	Kerkhof et al. (2010)	Residential address	Birth year	BC, R^2 = 81%<br>NO_2, R^2 = 85%<br>$PM_{2.5}$, R^2 = 73%
	Krämer et al. (2009)	Residential address<br>(**considering moving residence**)	Birth year, at 6 y.o.	BC, R^2 = 65%<br>NO_2, R^2 = 81%
	LeMasters et al. (2015)	Addresses of all locations where the child spent 8 or more hours in an average week from birth to age 7 y.o. (e.g., home, daycare, relative's home, school)	Time-weighted average	EC, R^2 = 74%
	MacIntyre et al. (2014)	Residential address	Birth year	Differs by cohort, all included in this table
	Mölter et al. (2014a)	The child's home (kitchen, living room, bedroom), the child's school and the journey between home and school	Birth year, birth to review (age 0–3, 0–5, 0–8, 0–11), 1 year prior to review (ages 2–3, 4–5, 7–8, 10–11)	BC, R^2 = 91%<br>NO_2, R^2 = 83%<br>NO_x, R^2 = 83%<br>$PM_{2.5}$, R^2 = 35%<br>PM_{10}, R^2 = 84%<br>PM_{coarse}, R^2 = 79%
	Mölter et al. (2014b)	Residential address	Birth year, age 4 or 5 years and age 8 or 10 years' addresses	NO_2, R^2 = 67% - 87%<br>NO_x, R^2 = 76% - 84%

	Morgenstern et al. (2007)	Residential address	Birth year	BC, R^2 = 47%<br>NO_2, R^2 = 51%<br>$PM_{2.5}$, R^2 =36%
	Morgenstern et al. (2008)	Residential address<br>(**considering moving residence**)	Birth year and at 2 or 3 y.o. and at 6 y.o.	BC, R^2 = 42%<br>NO_2, R^2 = 51%<br>$PM_{2.5}$, R^2 = 36%
	Ranzi et al. (2014)	Residential address<br>(**considering moving residence**)	Birth year, current exposure (calculated at residence 6 months before the date of interview), time-weighted average (from birth to 6 months before the interview)	NO_2, R^2 = 70%
	Tétreault et al. (2016)	Residential address<br>(**considering moving residence**)	Birth year, time-varying annual exposures	NO_2, R^2 = 80%
	Yang et al. (2016)	Residential address	Birth year, current year	BC, R^2= 92%<br>NO_2, R^2= 86%<br>$PM_{2.5}$, R^2= 67%
**Dispersion modelling**	Gruzieva et al. (2013)	Residential, daycare, and school addresses<br>(**unclear if they considered moving houses**)	Birth year, current exposure (12 months before the date of a questionnaire), average exposure during since the previous follow-up	NO_x, R^2 = 74% - 80%<br>PM_{10}, R^2 = 61%
	Hasunuma et al. (2016)	Residential and nursery address<br>(**considering moving residence**)	Time-weighted average during the first 1.5 years of life	EC, R^2 = 59% - 67%<br>NO_x, R^2 = 76% - 88%

	Lindgren et al. (2013)	Residential address<br>(**considering moving residence**)	Birth year, lifetime mean (2005-2010)	NO_x, R_s = 80%
	MacIntyre et al. (2014) – BAMSE cohort only	Residential address at birth	Birth year	NO_x, r^2 = 0.93
	McConnell et al. (2010)	Residential and school addresses<br>(**sensitivity analysis restricted to lifetime residents at same address**)	1997 (little change from 1997 over the lifetime of these children prior to study enrollment)	NO_2, R^2 = 34% - 56%
	Oftedal et al. (2009)	Residential address<br>(**considering moving residence**)	Birth year, cumulatively before onset	NO_2, r = 76%
	Rancière et al. (2016)	Residential and daycare address<br>(**considering moving residence**)	Birth year	NOx, r = 0.89
	Yamazaki et al. (2014)	Residential and school addresses	Two years mean before asthma development	EC, R^2= 59% - 67%<br>NO_x, R^2= 76% - 88%
**Residential measurements (NO_2)**	Jerrett et al. (2008)	Residential address	Annual/ fall-winter/ summer 2 weeks' average concentrations in 2000	No validation

**Remote sensing ($PM_{2.5}$)**	Tétreault et al. (2016)	Post code<br>(**considering moving residence**)	Birth year, time-varying annual exposures	No validation

References

BRAUER, M., HOEK, G., SMIT, H., DE JONGSTE, J., GERRITSEN, J., POSTMA, D. S., KERKHOF, M. & BRUNEKREEF, B. 2007. Air pollution and development of asthma, allergy and infections in a birth cohort. *European Respiratory Journal,* 29**,** 879-888.

BRAUER, M., HOEK, G., VAN VLIET, P., MELIEFSTE, K., FISCHER, P. H., WIJGA, A., KOOPMAN, L. P., NEIJENS, H. J., GERRITSEN, J. & KERKHOF, M. 2002. Air pollution from traffic and the development of respiratory infections and asthmatic and allergic symptoms in children. *American journal of respiratory and critical care medicine,* 166**,** 1092-1098.

BRUNST, K. J., RYAN, P. H., BROKAMP, C., BERNSTEIN, D., REPONEN, T., LOCKEY, J., KHURANA HERSHEY, G. K., LEVIN, L., GRINSHPUN, S. A. & LEMASTERS, G. 2015. Timing and duration of traffic-related air pollution exposure and the risk for childhood wheeze and asthma. *American journal of respiratory and critical care medicine,* 192**,** 421-427.

CARLSTEN, C., DYBUNCIO, A., BECKER, A., CHAN-YEUNG, M. & BRAUER, M. 2010. Traffic-related air pollution and incident asthma in a high-risk birth cohort. *Occupational and environmental medicine***,** oem-2010.

CLARK, N. A., DEMERS, P. A., KARR, C. J., KOEHOORN, M., LENCAR, C., TAMBURIC, L. & BRAUER, M. 2010. Effect of early life exposure to air pollution on development of childhood asthma. *Environmental Health Perspectives,* 118**,** 284.

DELL, S. D., JERRETT, M., BECKERMAN, B., BROOK, J. R., FOTY, R. G., GILBERT, N. L., MARSHALL, L., MILLER, J. D., TO, T. & WALTER, S. D. 2014. Presence of other allergic disease modifies the effect of early childhood traffic-related air pollution exposure on asthma prevalence. *Environment International,* 65**,** 83-92.

DENG, Q., LU, C., NORBÄCK, D., BORNEHAG, C.-G., ZHANG, Y., LIU, W., YUAN, H. & SUNDELL, J. 2015. Early life exposure to ambient air pollution and childhood asthma in China. *Environmental research,* 143**,** 83-92.

DENG, Q., LU, C., OU, C., CHEN, L. & YUAN, H. 2016. Preconceptional, prenatal and postnatal exposure to outdoor and indoor environmental factors on allergic diseases/symptoms in preschool children. *Chemosphere,* 152**,** 459-467.

ENGLISH, P., NEUTRA, R., SCALF, R., SULLIVAN, M., WALLER, L. & ZHU, L. 1999. Examining associations between childhood asthma and traffic flow using a geographic information system. *Environmental Health Perspectives,* 107**,** 761.

FUERTES, E., STANDL, M., CYRYS, J., BERDEL, D., VON BERG, A., BAUER, C.-P., KRÄMER, U., SUGIRI, D., LEHMANN, I. & KOLETZKO, S. 2013. A longitudinal analysis of associations between traffic-related air pollution with asthma, allergies and sensitization in the GINIplus and LISAplus birth cohorts. *PeerJ,* 1**,** e193.

GEHRING, U., BEELEN, R., EEFTENS, M., HOEK, G., DE HOOGH, K., DE JONGSTE, J. C., KEUKEN, M., KOPPELMAN, G. H., MELIEFSTE, K. & OLDENWENING, M. 2015a. Particulate matter composition and respiratory health: the PIAMA Birth Cohort Study. *Epidemiology,* 26**,** 300-309.

GEHRING, U., CYRYS, J., SEDLMEIR, G., BRUNEKREEF, B., BELLANDER, T., FISCHER, P., BAUER, C., REINHARDT, D., WICHMANN, H. & HEINRICH, J. 2002. Traffic-related air pollution and respiratory health during the first 2 yrs of life. *European Respiratory Journal,* 19**,** 690-698.

GEHRING, U., WIJGA, A. H., BRAUER, M., FISCHER, P., DE JONGSTE, J. C., KERKHOF, M., OLDENWENING, M., SMIT, H. A. & BRUNEKREEF, B. 2010. Traffic-related air pollution and the development of asthma and allergies during the first 8 years of life. *American Journal of Respiratory and Critical Care Medicine,* 181**,** 596-603.

GEHRING, U., WIJGA, A. H., HOEK, G., BELLANDER, T., BERDEL, D., BRÜSKE, I., FUERTES, E., GRUZIEVA, O., HEINRICH, J. & HOFFMANN, B. 2015b. Exposure to air pollution and development of asthma and rhinoconjunctivitis throughout childhood and adolescence: a population-based birth cohort study. *The Lancet Respiratory Medicine,* 3**,** 933-942.

GRUZIEVA, O., BERGSTRÖM, A., HULCHIY, O., KULL, I., LIND, T., MELÉN, E., MOSKALENKO, V., PERSHAGEN, G. & BELLANDER, T. 2013. Exposure to air pollution from traffic and childhood asthma until 12 years of age. *Epidemiology,* 24**,** 54-61.

HASUNUMA, H., SATO, T., IWATA, T., KOHNO, Y., NITTA, H., ODAJIMA, H., OHARA, T., OMORI, T., ONO, M. & YAMAZAKI, S. 2016. Association between traffic-related air pollution and asthma in preschool children in a national Japanese nested case–control study. *BMJ open,* 6**,** e010410.

JERRETT, M., SHANKARDASS, K., BERHANE, K., GAUDERMAN, W. J., KÜNZLI, N., AVOL, E., GILLILAND, F., LURMANN, F., MOLITOR, J. N. & MOLITOR, J. T. 2008. Traffic-related air pollution and asthma onset in children: a prospective cohort study with individual exposure measurement. *Environmental Health Perspectives,* 116**,** 1433-1438.

KERKHOF, M., POSTMA, D., BRUNEKREEF, B., REIJMERINK, N., WIJGA, A., DE JONGSTE, J., GEHRING, U. & KOPPELMAN, G. 2010. Toll-like receptor 2 and 4 genes influence susceptibility to adverse effects of traffic-related air pollution on childhood asthma. *Thorax,* 65**,** 690-697.

KIM, J., HAN, Y., SEO, S. C., LEE, J. Y., CHOI, J., KIM, K. H., WOO, S.-Y., KIM, E.-H., KWON, H.-J. & CHEONG, H. K. Association of carbon monoxide levels with allergic diseases in children. Allergy and Asthma Proceedings, 2016. OceanSide Publications, Inc, e1-e7.

KRÄMER, U., SUGIRI, D., RANFT, U., KRUTMANN, J., VON BERG, A., BERDEL, D., BEHRENDT, H., KUHLBUSCH, T., HOCHADEL, M. & WICHMANN, H.-E. 2009. Eczema, respiratory allergies, and traffic-related air pollution in birth cohorts from small-town areas. *Journal of Dermatological Science,* 56**,** 99-105.

LEMASTERS, G., LEVIN, L., BERNSTEIN, D. I., LOCKEY, S. D., LOCKEY, J. E., BURKLE, J., KHURANA HERSHEY, G. K., BRUNST, K. & RYAN, P. H. 2015. Secondhand smoke and traffic exhaust confer opposing risks for asthma in normal and overweight children. *Obesity,* 23**,** 32-36.

LINDGREN, A., STROH, E., BJÖRK, J. & JAKOBSSON, K. 2013. Asthma incidence in children growing up close to traffic: a registry-based birth cohort. *Environmental Health,* 12**,** 91.

LIU, W., HUANG, C., HU, Y., FU, Q., ZOU, Z., SUN, C., SHEN, L., WANG, X., CAI, J. & PAN, J. 2016. Associations of gestational and early life exposures to ambient air pollution with childhood respiratory diseases in Shanghai, China: A retrospective cohort study. *Environment international,* 92**,** 284-293.

MACINTYRE, E. A., BRAUER, M., MELÉN, E., BAUER, C. P., BAUER, M., BERDEL, D., BERGSTRÖM, A., BRUNEKREEF, B., CHAN-YEUNG, M., KLÜMPER, C., FUERTES, E., GEHRING, U., GREF, A., HEINRICH, J., HERBARTH, O., KERKHOF, M., KOPPELMAN, G. H., KOZYRSKYJ, A. L., PERSHAGEN, G., POSTMA, D. S., THIERING, E., TIESLER, C. M. T., CARLSTEN, C. & GROUP, T. S. 2014. GSTP1 and TNF gene variants and associations between air pollution and incident childhood asthma: the traffic, asthma and genetics (TAG) study. *Environmental health perspectives,* 122**,** 418-424.

MCCONNELL, R., ISLAM, T., SHANKARDASS, K., JERRETT, M., LURMANN, F., GILLILAND, F. & GAUDERMAN, J. 2010. Childhood incident asthma and traffic-related air pollution at home and school. *Environmental Health Perspectives,* 118**,** 1021.

MÖLTER, A., AGIUS, R., DE VOCHT, F., LINDLEY, S., GERRARD, W., CUSTOVIC, A. & SIMPSON, A. 2014a. Effects of long-term exposure to PM10 and NO2 on asthma and wheeze in a prospective birth cohort. *Journal of epidemiology and community health,* 68**,** 21-28.

MÖLTER, A., SIMPSON, A., BERDEL, D., BRUNEKREEF, B., CUSTOVIC, A., CYRYS, J., DE JONGSTE, J., DE VOCHT, F., FUERTES, E. & GEHRING, U. 2014b. A multicentre study of air pollution exposure and childhood asthma prevalence: the ESCAPE project. *European Respiratory Journal***,** erj00836-2014.

MORGENSTERN, V., ZUTAVERN, A., CYRYS, J., BROCKOW, I., GEHRING, U., KOLETZKO, S., BAUER, C.-P., REINHARDT, D., WICHMANN, H.-E. & HEINRICH, J. 2007. Respiratory health and individual estimated exposure to traffic-related air pollutants in a cohort of young children. *Occupational and environmental medicine,* 64**,** 8-16.

MORGENSTERN, V., ZUTAVERN, A., CYRYS, J., BROCKOW, I., KOLETZKO, S., KRAMER, U., BEHRENDT, H., HERBARTH, O., VON BERG, A. & BAUER, C. P. 2008. Atopic diseases, allergic sensitization, and exposure to traffic-related air pollution in children. *American Journal of Respiratory and Critical Care Medicine,* 177**,** 1331-1337.

OFTEDAL, B., NYSTAD, W., BRUNEKREEF, B. & NAFSTAD, P. 2009. Long-term traffic-related exposures and asthma onset in schoolchildren in Oslo, Norway. *Environmental Health Perspectives,* 117**,** 839-844.

PATEL, M. M., QUINN, J. W., JUNG, K. H., HOEPNER, L., DIAZ, D., PERZANOWSKI, M., RUNDLE, A., KINNEY, P. L., PERERA, F. P. & MILLER, R. L. 2011. Traffic density and stationary sources of air pollution associated with wheeze, asthma, and immunoglobulin E from birth to age 5 years among New York City children. *Environmental Research,* 111**,** 1222-1229.

RANCIÈRE, F., BOUGAS, N., VIOLA, M. & MOMAS, I. 2016. Early exposure to traffic-related air pollution, respiratory symptoms at 4 years of age, and potential effect modification by parental allergy, stressful family events, and gender: A prospective follow-up study of the PARIS birth cohort. *Environmental health perspectives*.

RANZI, A., PORTA, D., BADALONI, C., CESARONI, G., LAURIOLA, P., DAVOLI, M. & FORASTIERE, F. 2014. Exposure to air pollution and respiratory symptoms during the first 7 years of life in an Italian birth cohort. *Occupational and Environmental Medicine***,** oemed-2013-101867.

SHIMA, M. & ADACHI, M. 2000. Effect of outdoor and indoor nitrogen dioxide on respiratory symptoms in schoolchildren. *International Journal of Epidemiology,* 29**,** 862-870.

SHIMA, M., NITTA, Y. & ADACHI, M. 2003. Traffic-related air pollution and respiratory symptoms in children living along trunk roads in Chiba Prefecture, Japan. *Journal of Epidemiology,* 13**,** 108-119.

SHIMA, M., NITTA, Y., ANDO, M. & ADACHI, M. 2002. Effects of air pollution on the prevalence and incidence of asthma in children. *Archives of Environmental Health: An International Journal,* 57**,** 529-535.

TÉTREAULT, L.-F., DOUCET, M., GAMACHE, P., FOURNIER, M., BRAND, A., KOSATSKY, T. & SMARGIASSI, A. 2016. Childhood Exposure to Ambient Air Pollutants and the Onset of Asthma: An Administrative Cohort Study in Québec. *Environmental Health Perspectives*.

WANG, I.-J., TUNG, T.-H., TANG, C.-S. & ZHAO, Z.-H. 2016. Allergens, air pollutants, and childhood allergic diseases. *International Journal of Hygiene and Environmental Health,* 219**,** 66-71.

YAMAZAKI, S., SHIMA, M., NAKADATE, T., OHARA, T., OMORI, T., ONO, M., SATO, T. & NITTA, H. 2014. Association between traffic-related air pollution and development of asthma in school children: cohort study in Japan. *Journal of Exposure Science and Environmental Epidemiology,* 24**,** 372-379.

YANG, A., JANSSEN, N. A., BRUNEKREEF, B., CASSEE, F. R., HOEK, G. & GEHRING, U. 2016. Children's respiratory health and oxidative potential of PM2. 5: the PIAMA birth cohort study. *Occupational and environmental medicine***,** oemed-2015-103175.

ZMIROU, D., GAUVIN, S., PIN, I., MOMAS, I., SAHRAOUI, F., JUST, J., LE MOULLEC, Y., BREMONT, F., CASSADOU, S. & REUNGOAT, P. 2004. Traffic related air pollution and incidence of childhood asthma: results of the Vesta case-control study. *Journal of Epidemiology and Community Health,* 58**,** 18-23.
